# Appropriate duration of peripherally inserted central catheter maintenance to prevent central line-associated bloodstream infection

**DOI:** 10.1371/journal.pone.0234966

**Published:** 2020-06-22

**Authors:** Seonghun Park, Shinje Moon, Hyunjoo Pai, Bongyoung Kim

**Affiliations:** 1 School of Medicine, Hanyang University College of Medicine, Seoul, Korea; 2 Department of Internal Medicine, Hallym University College of Medicine, Chuncheon, Korea; 3 Department of Internal Medicine, Hanyang University College of Medicine, Seoul, Korea; University Magna Graecia of Catanzaro, ITALY

## Abstract

**Background/aim:**

Prolonged maintenance of central venous catheters, including peripherally inserted central catheters (PICCs), is a major risk factor for central line-associated bloodstream infection (CLABSI). This study was conducted to evaluate the appropriate duration of PICC maintenance to prevent CLABSI.

**Methods:**

A single-center retrospective study was conducted at an 824-bed tertiary hospital in Korea between January 2010 and December 2017. All hospitalized patients who underwent ultrasound-guided PICC insertion were enrolled. CLABSI was diagnosed according to the definitions of the National Health Safety Network. CLABSI caused by PICC was defined as PICC-associated bloodstream infection (PABSI). To identifying statistical correlations between catheter days and PABSI, the odds ratio for PABSI on the basis of the continuous value of catheter days was analyzed using restricted cubic spline splits with five knots. The optimal cut-off value for catheter days was identified by maximizing the area under the receiver operating characteristic (ROC) curve (AUC).

**Results:**

A total of 1,053 patients underwent ultrasound-guided PICC insertion during the study period. Among them, 36 were confirmed as having a PABSI (3.5%, 36/1014; 1.14 per 1000 catheter days). In the restricted cubic spline regression, catheter days showed a dose-dependent relationship with the risk of PABSI. The AUC of the ROC curve for developing a PABSI according to the duration of catheter maintenance was 0.715 (95% CI, 0.639–0.790); the calculated optimal cut-off value was 25 days.

**Conclusion:**

The incidence of PABSI was 1.14 per 1000 catheter days and the optimal cut-off value of catheter days to avoid a PABSI was 25 days.

## Introduction

A peripherally inserted central catheter (PICC) is a catheter that is inserted in a peripheral vein in the arm with its tip resting in the superior vena cava [1]. The use of PICCs has increased markedly, as they allow non-permanent and durable venous access for the delivery of antibiotics, chemotherapy, and total parenteral nutrition [2]. PICC use was introduced in Korean hospitals recently and has grown rapidly since the 2000s because of the increase in the number of elderly patients with difficult vascular access. Currently, it is not difficult to find patients with PICCs in Korean hospitals, including long-term care hospitals. However, the use of PICCs can cause some complications, including central line-associated bloodstream infection (CLABSI), which increases mortality, morbidity, and medical costs [3].

A major risk factor for CLABSI is the prolonged maintenance of central venous catheters (CVCs) [4]. However, the exact duration for which CVCs can be maintained without causing CLABSI has not been determined [5]. Instead, it is recommended that CVCs should be replaced by cuffed tunneled catheters at a subcutaneous venous port to reduce catheter-associated complications in the case of prolonged use of CVCs without a cuff [6]. In the real world setting, maintaining CVCs, including PICCs, in hospitalized patients for more than three weeks is common [7, 8].

This study was conducted to analyze the clinical characteristics of patients with PICC-associated bloodstream infection (PABSI) and to evaluate the appropriate duration of PICC maintenance to avoid CLABSI.

## Material and methods

### Study setting

A single-center retrospective study was conducted in an 824-bed tertiary hospital in Korea between January 2010 and December 2017. All hospitalized patients who underwent ultrasound-guided PICC insertion were enrolled. Patients were excluded if they (1) were under 19 years old, (2) had died, were discharged, or were transferred to other medical institutions within 3 days of PICC placement, or (3) had at least one test result for absolute neutrophil count (ANC) under 500 cells/μL during catheterization.

### Definitions

CLABSI was diagnosed using the following definition from the National Health Safety Network (NHSN): a laboratory-confirmed bloodstream infection in patients wherein an eligible bloodstream infection-causing organism was identified and an eligible CVC was present on or one day before the infection date [1]. A laboratory-confirmed bloodstream infection is defined as one that involves: (1) a bacterial or fungal pathogen that is not a common commensal organism according to the NHSN definition, detected from one or more blood specimens and unrelated to infections at other sites or (2) having at least one of the following symptoms: fever (>38°C), chills, or hypotension, along with the identification of an organism(s) in the blood that is unrelated to infection at other sites and isolation of common commensal organisms, according to the NHSN definition, from two or more blood specimens collected on separate occasions [1]. Patients who had other types of CVCs in addition to a PICC were excluded from analyses as it was difficult to identify the culprit catheter. Finally, after exclusion, the remaining patients were diagnosed with PABSI.

We defined catheter days as the duration for which patients could maintain a PICC without developing a PABSI. If patients were discharged home or transferred to other medical institutions without removal of the PICC and identification of the exact date of PICC removal was impossible, we considered the day of discharge as the catheter removal day.

Empirical antibiotics were considered effective if one or more antibiotics that were administered to the patients within 48 hours from diagnosis of PABSI were found to be active against the causative organism on the basis of *in vitro* susceptibility testing and if the dose and route of administration conformed to current medical standards [2].

The hopeless discharge was defined as the cases when a patient with almost no chance of recovery discharge from the hospital. When an episode's treatment result was recorded as 'hopeless discharge', the clinical outcome of the case was classified as 'hopeless discharge'.

Early and late infections were classified according to the day of occurrence of the PABSI. We defined early and late infection as infection within 30 days and after 30 days from the day of catheter insertion, respectively.

### Data collection

To identify the appropriate duration for maintaining a PICC, we collected data on catheter days from patients who were not diagnosed with CLABSI and PABSI. From patients with PABSI, we collected the following information: (1) demographic data (age, sex, and body mass index [BMI]), (2) underlying diseases (diabetes mellitus, peripheral vascular disease, any malignancy, chronic kidney disease, chronic pulmonary disease, liver cirrhosis, heart disease, neurological disease, rheumatic disease, and Charlson’s comorbidity index [3]), (3) risk factors for PABSI (catheter insertion during intensive care unit admission, catheter days, lipid-containing parenteral nutrition days, other catheter-associated complications, use of immunosuppressants, chemotherapy, and use of any antibiotic within one week before the diagnosis of PABSI), (4) laboratory findings on the catheter insertion day (white blood cell count, creatinine, albumin, and c-reactive protein levels), (5) antibiotic prescription, (6) microorganisms isolated from blood culture, and (7) clinical outcomes.

### Statistical analysis

To identify any statistical correlation between catheter days and PABSI, the odds ratio (OR) for PABSI according to the number of catheter days was analyzed using restricted cubic spline splits with five knots. A receiver operating characteristic (ROC) curve was used to determine a proper catheter days to avoid PABSI. The optimal cut-off value of catheter days was identified by maximizing the area under the ROC curve (AUC).

Categorical variables were analyzed using the chi-square test or Fisher’s exact test, as appropriate. Continuous variables were analyzed using the Mann-Whitney *U* test or independent *t*-tests.

Analyses were carried out using the statistical package, R (version 3.3.2, R Foundation for Statistical Computing). The significance level was set at *P* < 0.05.

### Ethics statement

The study protocol was approved by the institutional review boards of Hanyang University Seoul Hospital (IRB number: 2018-05-035), and the requirement for written informed consent from the patients was waived because of the retrospective nature of the study.

## Results

### Characteristics of patients with PABSI

**Fig 1:**
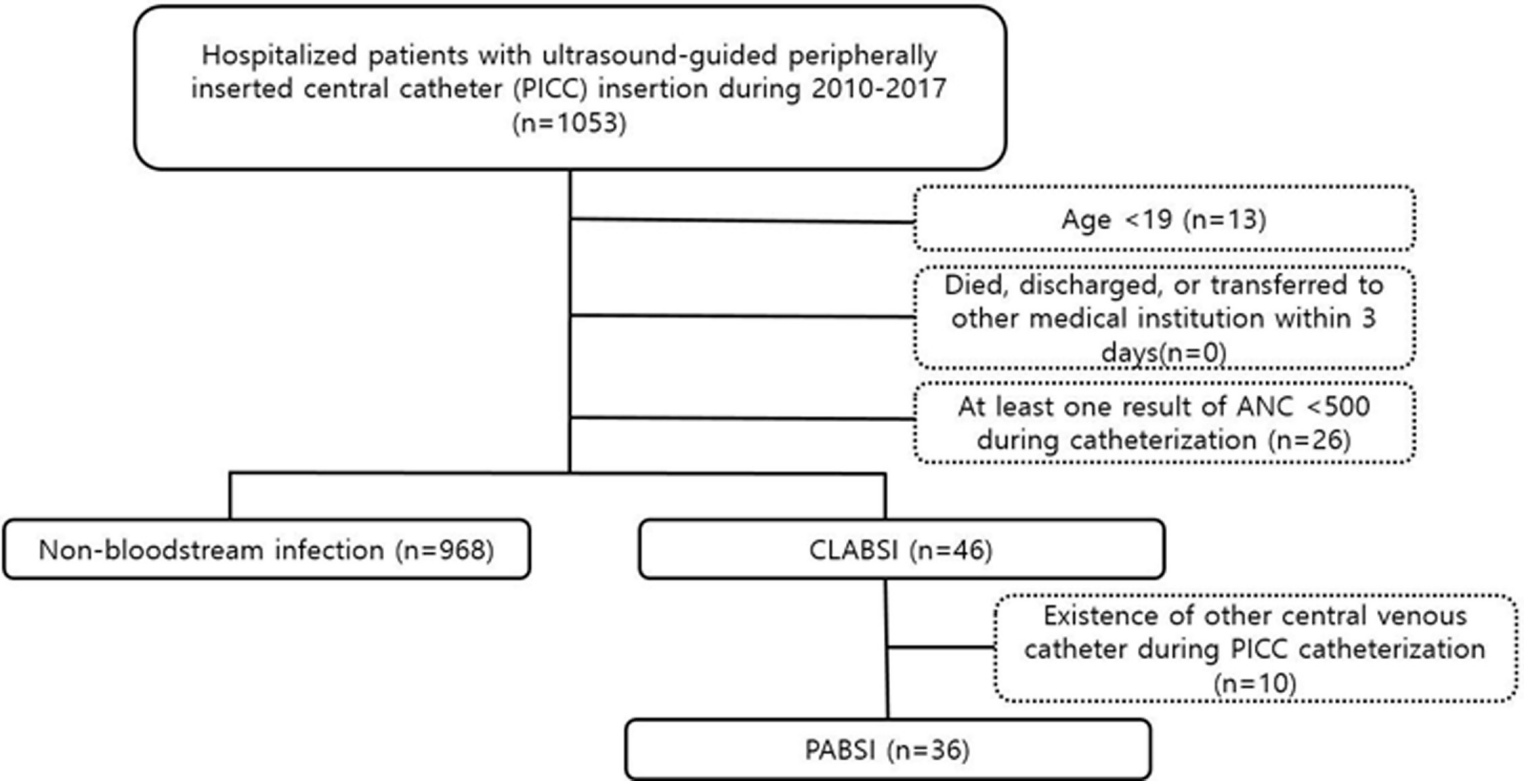
Flow diagram showing the process for selecting cases in this study.

A total of 1,053 patients underwent ultrasound-guided PICC insertion during the study period. Among them, 39 patients were excluded because of the following reasons: 13 were aged under 19 years and 26 had an ANC less than 500 cells/μL. A total of 46 patients were confirmed as having CLABSI, 10 of whom had other types of CVCs in addition to PICCs. Finally, 36 cases were confirmed as having PABSI (Fig 1). Accordingly, the incidence of PABSI was calculated as 1.14 per 1000 catheter days (3.5%, 36/1014).

Table 1 summarizes the baseline clinical characteristics of the patients with PABSI. The median age was 74.5 years (interquartile range [IQR] 57.5–84), and 25% of patients (9/36) were male. Regarding underlying diseases, the median Charlson’s comorbidity index was 3 (2–4.75) and 52.8% of patients (19/36) had neurological diseases, which were the most frequent comorbidity, followed by malignancy (44.4%, 16/36), diabetes mellitus (30.6%, 11/36), and heart disease (30.6%, 11/36).

**Table 1:** Clinical characteristics of patients with peripherally inserted central catheter-associated bloodstream infections.

Clinical parameters	Total (n = 36)
Demographic data	
Age, median (IQR)	74.5 (57.5–84)
Male sex (%)	9 (25.0)
BMI, kg/m^2^, mean ± SD	22.08 ± 3.98
Underlying disease	
Charlson’s comorbidity index, median (IQR)	3 (2–4.75)
Neurologic disease (%)	19 (52.8)
Any malignancy (%)	16 (44.4)
Diabetes mellitus (%)	11 (30.6)
Heart disease (%)	11 (30.6)
Rheumatic disease (%)	6 (16.7)
Peripheral vascular disease (%)	5 (13.9)
Chronic kidney disease (%)	2 (5.6)
Chronic pulmonary disease (%)	3 (8.3)
Risk factors for CLABSI	
Use of any antibiotics within one week before the diagnosis (%)	22 (61.1)
Use of immunosuppressant (%)	7 (19.4)
Catheter insertion during ICU admission (%)	3 (8.3)
Use of chemotherapeutic agents (%)	1 (2.8)
Laboratory findings on catheter insertion day	
White blood cell count, 10^3^ cells/mm^3^, mean ± SD	11.10 ± 19.45
Creatinine, mg/dL, mean ± SD	0.91 ± 0.83
Albumin, g/dL, mean ± SD	3.22 ± 0.64
C-reactive protein, mg/dL, mean ± SD	7.04 ± 8.92
Duration of catheter maintenance, median (IQR)	41 (30.25–61)
Duration of parenteral nutrition with lipid-containing formula, median (IQR)	22 (9–35)
Concordant empirical antibiotics (%)	17 (47.2)
Clinical outcomes	
Recovery (%)	24 (66.7)
30-day mortality (%)	10 (27.8)
Hopeless discharge (%)	2 (5.6)

Abbreviations: IQR, interquartile range; SD, standard deviation; BMI, body mass index; CLABSI, central line-associated bloodstream infection; ICU, intensive care unit

No patients had other catheter-associated complications, such as phlebitis, thrombosis, or catheter malposition. One-third of patients died within 30 days or were discharged home without hope of recovery.

The most commonly isolated microorganism was coagulase-negative *Staphylococci* (41.7%, 15/36), followed by non-albicans *Candida* spp. (25.0%, 9/36) and *Candida albicans* (16.7%, 6/36) (Table 2).

**Table 2:** Microorganisms isolated from blood culture among patients with peripherally inserted central catheter-associated bloodstream infection.

Microorganisms	Total (n = 36)
Gram-positive bacteria	
Coagulase-negative *Staphylococci* (%)	15 (41.7)
*Enterococcus* spp. (%)	2 (5.6)
*Staphylococcus aureus* (%)	1 (2.8)
Gram-negative bacteria	
*Acinetobacter baumanii* (%)	2 (5.6)
*Klebsiella pneumoniae* (%)	1 (2.8)
Fungi	
*Candida albicans* (%)	6 (16.7)
Other *Candida* spp. (%)	9 (25.0)

### Comparison of clinical characteristics between early and late infections

Table 3 summarizes the clinical characteristics of patients with PABSI according to the day of the occurrence of PABSI. Overall, there was no significant difference in clinical characteristics, including age, sex, underlying diseases, risk factors for CLABSI, initial laboratory findings, microorganisms, or clinical outcomes, between the two groups.

**Table 3:** Comparison of clinical characteristics between early and late infections.

	Early infection (n = 13)	Late infection (n = 23)	*P*-value
Demographic data			
Age, median (IQR)	75 (45–84.5)	74 (62–84)	0.820
Male sex (%)	2 (15.4)	7 (30.4)	0.438
BMI, kg/m^2^, mean ± SD	20.79 ± 3.44	22.82 ± 4.14	0.143
Underlying disease			
Charlson’s comorbidity index, median (IQR)	5 (2.5–6.5)	6 (4–6)	0.226
Neurologic disease (%)	5 (38.5)	14 (60.9)	0.196
Any malignancy (%)	4 (30.8)	12 (52.2)	0.214
Diabetes mellitus (%)	4 (30.8)	7 (30.4)	1.000
Heart disease (%)	2 (15.4)	9 (39.1)	0.259
Rheumatic disease (%)	4 (30.8)	2 (8.7)	0.161
Peripheral vascular disease (%)	0 (0)	5 (21.7)	0.136
Chronic kidney disease (%)	2 (15.4)	0 (0)	0.124
Chronic pulmonary disease (%)	0 (0)	3 (13.0)	0.288
Risk factors for CLABSI			
Use of any antibiotics within one week before the diagnosis (%)	8 (61.5)	14 (60.9)	0.968
Use of immunosuppressant (%)	4 (30.8)	3 (13.0)	0.225
Catheter insertion during ICU admission (%)	2 (15.4)	1 (4.3)	0.539
Use of chemotherapeutic agents (%)	0 (0)	1 (4.3)	1.000
Laboratory findings			
White blood cell count, 10^3^ cells/mm^3^, mean ± SD	15.41 ± 31.79	8.66 ± 5.78	0.324
Creatinine, mg/dL, mean ± SD	0.84 ± 0.76	0.94 ± 0.89	0.735
Albumin, g/dL, mean ± SD	3.23 ± 5.29	3.22 ± 0.70	0.980
C-reactive protein, mg/dL, mean ± SD	8.44 ± 12.40	6.24 ± 6.39	0.486
Microorganisms			
Gram-positive bacteria			
Coagulase-negative *Staphylococci* (%)	5 (38.5)	10 (43.5)	0.769
*Enterococcus* spp. (%)	1 (7.7)	1 (4.3)	1.000
*Staphylococcus aureus* (%)	0 (0)	1 (4.3)	1.000
Gram-negative bacteria			
*Acinetobacter baumanii* (%)	1 (7.7)	1 (4.3)	1.000
*Klebsiella pneumoniae* (%)	0 (0)	1 (4.3)	1.000
Fungi			
*Candida albicans* (%)	2 (15.4)	4 (17.4)	1.000
Other *Candida* spp. (%)	4 (30.8)	5 (21.7)	0.693
Concordant empirical antibiotics (%)	5 (38.5)	12 (52.2)	0.429
Clinical outcomes			
Recovery (%)	9 (69.2)	15 (65.2)	1.000
30-day mortality (%)	3 (23.1)	7 (30.4)	0.716
Hopeless discharge (%)	1 (7.7)	1 (4.3)	1.000

Abbreviations: IQR, interquartile range; SD, standard deviation; BMI, body mass index; CLABSI, central line-associated bloodstream infection; ICU, intensive care unit

### Appropriate maintenance duration for PICC

**Fig 2:**
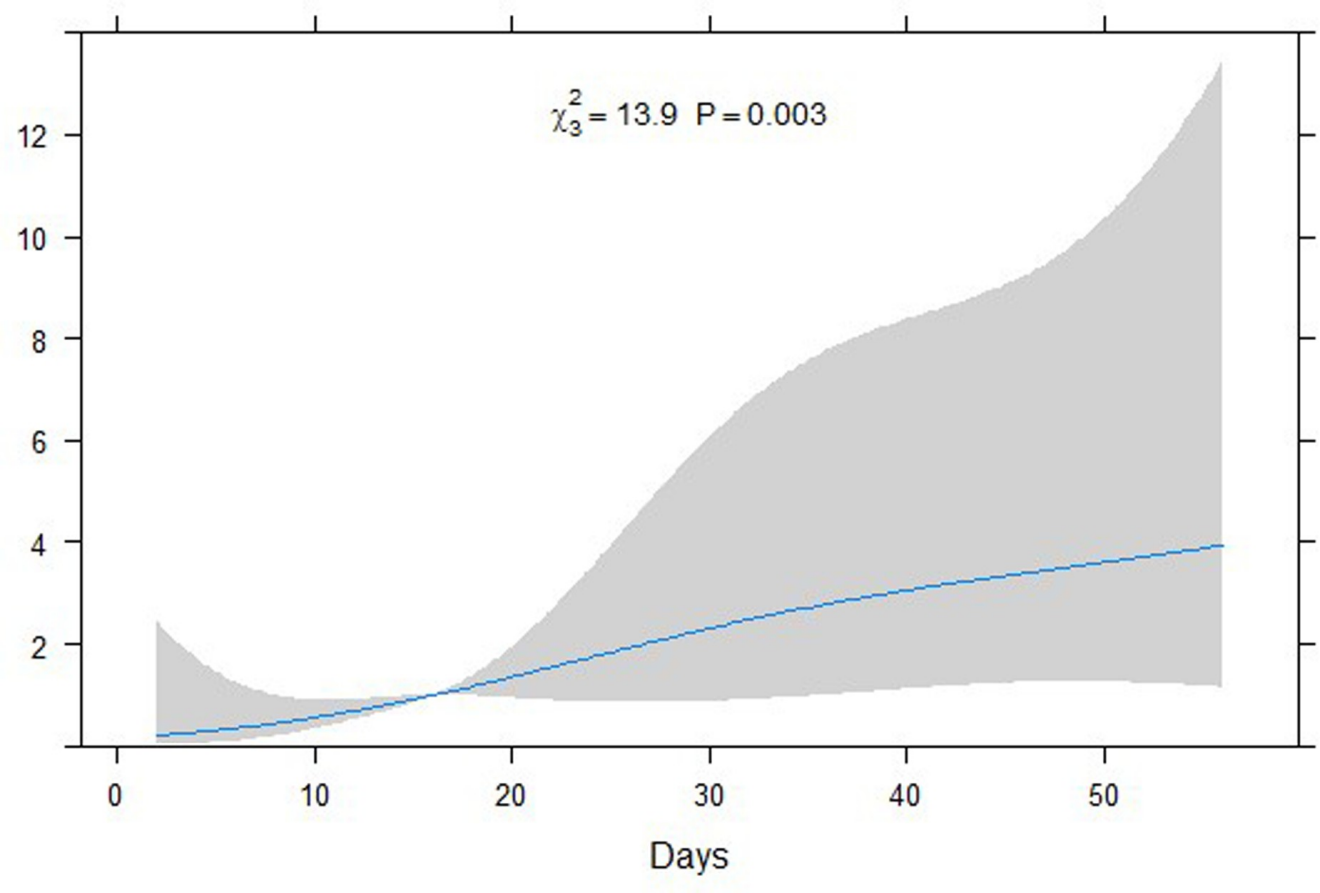
Odds ratio for peripherally inserted central catheter-associated bloodstream infection according to the number of catheter days.

**Fig 3:**
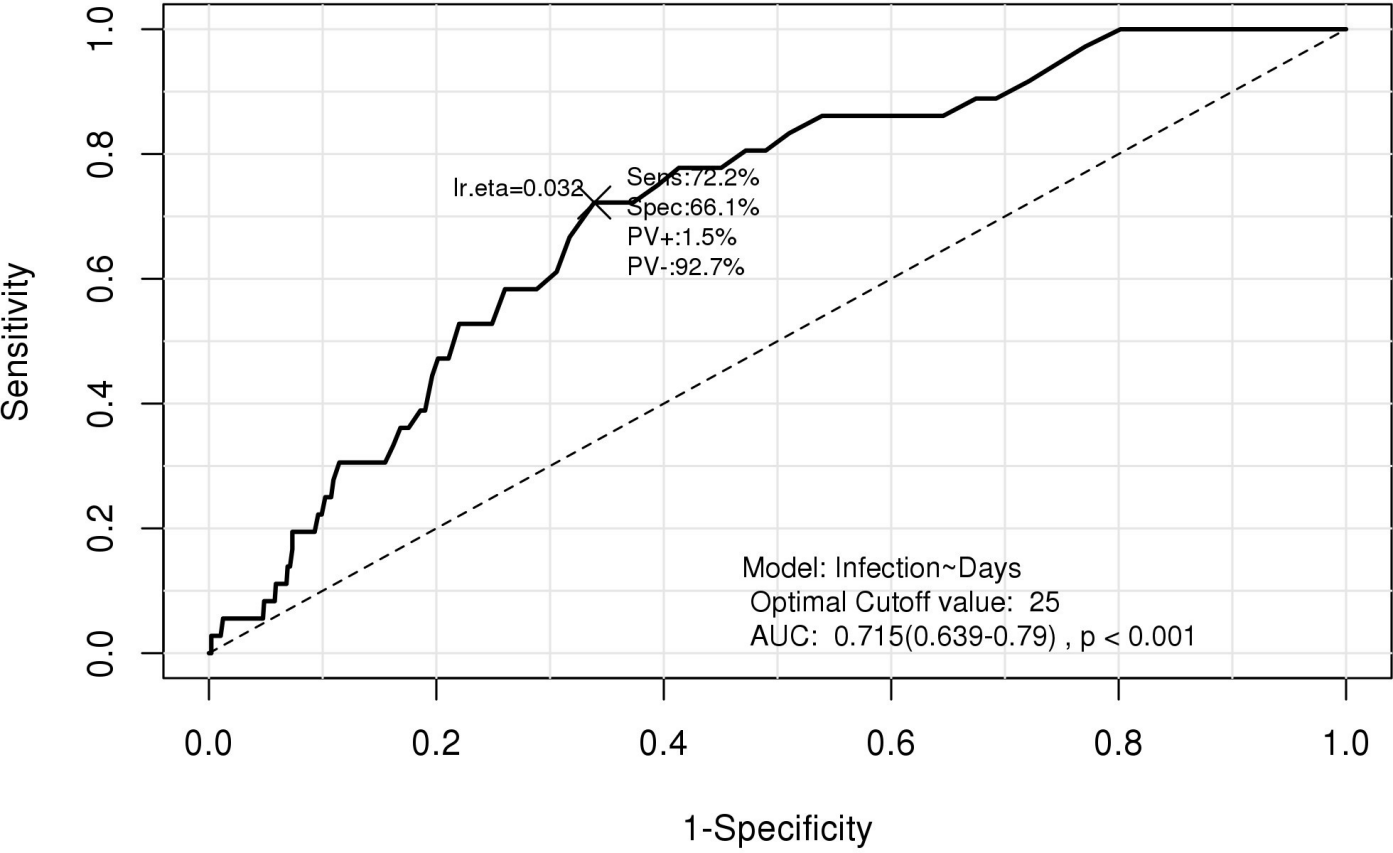
Receiver operating characteristics curves for peripherally inserted central catheter-associated bloodstream infection on the basis of the number of catheter days.

In the restricted cubic spline regression, catheter days showed a dose-dependent relationship with the risk of PABSI (Fig 2). The ROC for developing PABSI according to catheter maintenance duration is presented in Fig 3. The AUC was 0.715 (95% CI, 0.639–0.790) and the optimal cut-off value was 25 days.

Table 4 presents data on the sensitivity, specificity, positive predictive value (PPV), and negative predictive value (NPV) for PABSI at different cut-off values of catheter days. The sensitivity, specificity, PPV, and NPV for the optimal cut-off value (25 days) were 88.9%, 32.5%, 4.7%, and 98.7%, respectively. During the occurrence of PABSI, 26 patients had had a PICC for less than 25 days.

**Table 4:** Sensitivity, specificity, positive predictive value, and negative predictive value for peripherally inserted central catheter-associated bloodstream infection at different cut-off values of catheter days.

Cut-off values of catheter days	No. of cases under the cut-off value	Sensitivity, %	Specificity, %	PPV, %	NPV, %
10	32/36	88.9	32.5	4.7	98.7
20	28/36	77.8	57.0	6.3	98.6
25	26/36	72.2	66.1	7.3	98.5
30	21/36	58.3	74.0	7.7	97.9
40	13/36	36.1	82.4	7.1	97.2
50	11/36	30.6	87.9	8.6	97.1

Abbreviations: PPV, positive predictive value; NPV, negative predictive value

## Discussion

The use of PICCs has increased significantly in the recent years because of several advantages, such as the lower risk of mechanical complications, relative ease of insertion, and increased patient tolerance compared to that for other CVCs [1, 9].

In addition to providing durable vascular access, PICCs have been perceived as being associated with a low risk of bloodstream infections, which may have contributed to their prolonged use [10]. Many hypotheses, including a lower bacterial burden and ease of site care in the skin over the arm, support the viewpoint that PICCs are safer than conventional CVCs in terms of bloodstream infections [11]. However, several studies have raised questions about the safety of PICCs in terms of bloodstream infections. According to a prospective cohort study in the US, PICC use in high-risk hospitalized patients resulted in a similar bloodstream infection rate as that caused by conventional CVCs [11]. Furthermore, a systematic review and meta-analysis revealed that the bloodstream infection rate was similar between hospitalized patients with PICCs and those with other types of CVCs [12]. Regardless of the controversies, applying appropriate strategies for preventing bloodstream infections in patients with PICCs is indispensable as these infections can have fatal consequences.

The known pathogens that most commonly cause CLABSI associated with non-cuffed catheters are coagulate-negative *Staphylococci*, *S*. *aureus*, *Candida* spp., and enteric gram-negative bacilli [13]. Because the insertion site and hub are prominent sources of microbes, the normal flora of the skin at the insertion site are often associated with CLABSI [14]. Similar to the results of studies in other countries, a recent retrospective study revealed that coagulase-negative *Staphylococci* (26.0%) and *S*. *aureus* (26.0%) were the most common pathogens in patients with CLABSI in a Korean hospital [7]. In the same study, the proportions of gram-negative bacilli and *Candida* spp. were 34.5% and 10.3%, respectively [7]. The distribution of pathogens causing PABSI does not seem to differ from that of pathogens causing CLABSI. A retrospective study in the US found that the proportion of coagulase-negative *Staphylococci* was 40.0%, which was the highest, and that of gram-negative bacilli and *Candida* spp. was 24.0% and 22.0%, respectively [10]. This result is similar to that of our study.

As for the duration of catheter maintenance, current guidelines recommend that catheter removal is required only if it is no longer needed [5]. On the basis of this recommendation, many patients who have limited vascular access and need prolonged parenteral therapy have non-tunneled catheters, including PICCs, inserted for periods longer than three weeks. We found the median duration of PICC maintenance among patients with PABSI to be 41 days. Therefore, identification of the duration after which the PICC should be removed in order to prevent PABSI is important and will aid clinicians in making decisions regarding the maintenance/removal of a catheter. More well-designed studies should be performed to validate our result, according to which the optimal duration of PICC maintenance is 25 days.

The current guideline does not recommend routine replacement of a catheter for the prevention of bloodstream infections [5, 15]. There are controlled studies that support this recommendation. A prospective randomized study showed that there was no difference in the risk of CLABSI between patients undergoing scheduled catheter replacement every 7 days and a control group [16]. In another controlled trial, CVCs were replaced every 3 days in the patient group, and no statistical difference in the rate of CLABSI between the patient group and control group was found [17]. However, it is debatable whether the results of studies conducted approximately 30 years ago could be applied directly to the current situation. According to the study by Cobb et al [17], the recommended mean duration of catheter maintenance is 10–17 days, which is much shorter than our finding. As the hospital environment, including medical practice and patient population, has changed significantly, this issue needs to be revisited.

There are some limitations to the present study. First, this study was conducted in a large university-affiliated tertiary care hospital. Therefore, the patients enrolled in the study do not represent the entire population of patients with PICCs, and the results cannot be generalized. Second, most patients in the study were inpatients with several comorbidities. Unlike that in other countries, outpatient parenteral therapy is not popular in South Korea and the majority of PICC insertions are performed for inpatients with complex vascular access. Third, other complications of PICCs and medical costs were not considered while calculating the appropriate duration of PICC maintenance. Finally, the characteristics of the patients with PABSI was not compared with that of the general population.

In conclusion, the incidence of PABSI was 1.14 per 1000 catheter days, and the optimal cut-off value of catheter days for preventing PABSIs was 25 days. Further research is necessary to evaluate the effectiveness of setting an exact duration for PICC maintenance.

## Supporting information

S1 Data (XLSX)

## Abbreviations

PICC: peripherally inserted central catheter
CLABSI: central line-associated bloodstream infection
CVC: central venous catheter
PABSI: PICC-associated bloodstream infection
ANC: absolute neutrophil count
NHSN: National Health Safety Network
BMI: body mass index
ROC: receiver operating characteristic
AUC: area under the ROC curve
IQR: interquartile range
PPV: positive predictive value
NPV: negative predictive value
